# ICTV Virus Taxonomy Profile: *Virgaviridae*

**DOI:** 10.1099/jgv.0.000884

**Published:** 2017-08-08

**Authors:** Michael J. Adams, Scott Adkins, Claude Bragard, David Gilmer, Dawei Li, Stuart A. MacFarlane, Sek-Man Wong, Ulrich Melcher, Claudio Ratti, Ki Hyun Ryu, ICTV Report Consortium

**Affiliations:** ^1^​ ICTV, Stevenage, Hertfordshire SG2 8BT, UK; ^2^​ USDA ARS USHRL, Fort Pierce, FL 34945, USA; ^3^​ Université Catholique de Louvain, Louvain-la-Neuve, Belgium; ^4^​ Institut de Biologie Moléculaire des Plantes, 67084 Strasbourg cedex, Strasbourg, France; ^5^​ State Key Laboratory for Agro-biotechnology, China Agricultural University, Beijing 100193, PR China; ^6^​ The James Hutton Institute, Dundee DD2 5DA, UK; ^7^​ Department of Biological Sciences, National University of Singapore, Singapore 117543, Singapore; ^8^​ Department of Biochemistry and Molecular Biology, Oklahoma State University, Stillwater, OK 74078, USA; ^9^​ Dipartimento di Scienze e Tecnologie Agroambientali, Università di Bologna, Bologna 40127, Italy; ^10^​ Department of Horticultural Science, Seoul Women's University, Seoul, Republic of Korea

**Keywords:** *Virgaviridae*, ICTV, taxonomy, tobacco mosaic virus

## Abstract

The family *Virgaviridae* is a family of plant viruses with rod-shaped virions, a ssRNA genome with a 3′-terminal tRNA-like structure and a replication protein typical of alpha-like viruses. Differences in the number of genome components, genome organization and the mode of transmission provide the basis for genus demarcation. Tobacco mosaic virus (genus *Tobamovirus*) was the first virus to be discovered (in 1886); it is present in high concentrations in infected plants, is extremely stable and has been extensively studied. This is a summary of the International Committee on Taxonomy of Viruses (ICTV) Report on the taxonomy of the *Virgaviridae*, which is available at www.ictv.global/report/virgaviridae.

## Abbreviation

ICTV, International Committee on Taxonomy of Viruses.

## Virion

**Fig. 1.**
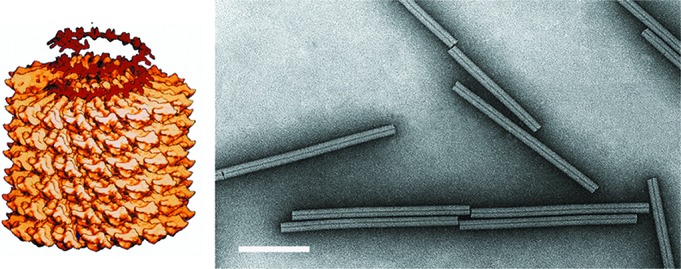
(Left) Model of a particle of tobacco mosaic virus. Also shown is the RNA as it is thought to participate in the assembly process. (Right) Negative contrast electron micrograph of tobacco mosaic virus particles stained with uranyl acetate. Bar, 100 nm.

The non-enveloped, rod-shaped virus particles of members of the family *Virgaviridae* are helically constructed with a pitch of 2.3 to 2.5 nm and an axial canal (Table 1, Fig. 1). They are about 20 nm in diameter, with predominant lengths that depend upon the genus. In most viruses, the capsid comprises multiple copies of a single protein of about 17–24 kDa [1]. In viruses of the genera *Furovirus* and *Pomovirus* (all transmitted by plasmodiophorids), a larger minor capsid protein is also produced by translational readthrough of the capsid protein-encoding gene stop codon and can be detected at the extremity of virus particles [2]. In at least some furoviruses, a further minor coat protein of 25 kDa is initiated from a CUG codon upstream of the canonical start codon [3].

## Genome

**Fig. 2.**
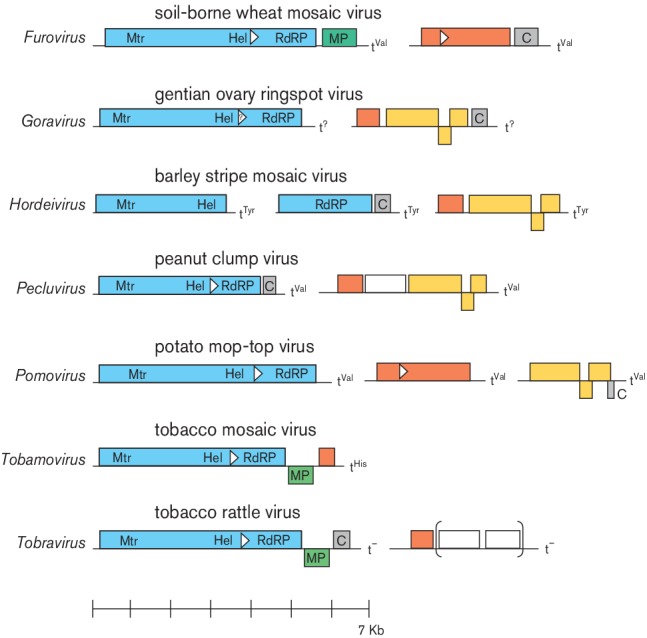
Genome organization of representative viruses from each genus in the family *Virgaviridae*. Colours indicate replication proteins (blue) with methyltransferase (Mtr), helicase (Hel) and RNA-dependent RNA polymerase (RdRP) domains marked; movement proteins (MP) of the 30K superfamily (green) and triple gene block proteins (yellow); coat proteins (orange); cysteine-rich proteins (grey); and other proteins (white). White triangular arrowheads show the positions of suppressible stop codons that result in larger, readthrough products. tRNA-like structures at the 3′ termini of the genomic RNAs are also shown. Brackets indicate ORFs not present in all isolates.

The positive-sense ssRNA genome has a 5′-cap (m^7^GpppG) and a 3′-terminal tRNA-like structure that accepts histidine (*Tobamovirus*), tyrosine (*Hordeivirus*) or valine (*Furovirus*, *Pecluvirus*, *Pomovirus*). The number of genome components depends upon the genus (Fig. 2). The largest ORF encodes a replication protein with conserved methyltransferase and helicase domains, an arrangement typical of alpha-like viruses. This protein is translated directly from the genomic RNA. In viruses of all genera except *Hordeivirus*, the RNA-dependent RNA polymerase is expressed as the C-terminal part of this protein by readthrough of a leaky stop codon. All viruses encode cell-to-cell movement proteins which, depending on the genus, are either single proteins of the ‘30K’-type or a 'triple gene block'.

## Replication

Tobamovirus RNA replication occurs via several steps: (a) synthesis of viral replication proteins by translation of the genomic RNA; (b) translation-coupled binding of the replication proteins to a 5′-terminal region of the genomic RNA; (c) recruitment of the genomic RNA by replication proteins onto membranes and formation of a complex with host proteins TOM1 and ARL8; (d) synthesis of complementary (negative-strand) RNA in the complex; and (e) synthesis of progeny genomic RNA [4].

## Taxonomy

There are seven genera with distinct genome organisations (Fig. 2) and other features as follows:

- *Goravirus.* Pollen transmission.
- *Furovirus.* Transmitted to graminaceous plants by the plasmodiophorid *Polymyxa graminis*. Soil-borne wheat mosaic virus is the best-known member.
- *Hordeivirus.* Pollen and seed transmission. Barley stripe virus is the best known member.
- *Pecluvirus.* Transmitted by the plasmodiophorid *Polymyxa graminis*.
- *Pomovirus.* Transmitted by plasmodiophorids.
- *Tobamovirus.* No natural vector. This large genus includes tobacco mosaic virus, the first virus to be discovered and crystalized, and since widely studied [5, 6].
- *Tobravirus.* Nematode transmission. Tobacco rattle virus is the best-known member.

The only plant viruses with rod-shaped particles not included in the family are those classified in the genus *Benyvirus*, family *Benyviridae*. Benyviruses have polyadenylated RNAs and replication proteins only distantly related to those of viruses in the family *Virgaviridae*.

## Resources

Full ICTV Online (10th) Report: www.ictv.global/report/virgaviridae.

**Table 1.** Characteristics of the family *Virgaviridae*

Typical member:	tobacco mosaic virus variant 1 (V01408), species *Tobacco mosaic virus*, genus *Tobamovirus*
Virion	Non-enveloped, rod-shaped particles about 20 nm in diameter and up to about 300 nm long. Except in members of the genus *Tobamovirus*, the particle length distribution is bi- or tri-modal
Genome	6.3 to 13 kb of positive-sense RNA; non-segmented in members of the genus *Tobamovirus*, but multipartite in other genera with segments separately encapsidated in 2 or 3 components
Replication	Cytoplasmic, probably associated with the endoplasmic reticulum
Translation	From full-length genomic or subgenomic mRNAs
Host Range	Plants
Taxonomy	Seven genera containing about 60 species
